# Predominant *porB1A *and *porB1B *genotypes and correlation of gene mutations with drug resistance in *Neisseria gonorrhoeae *isolates in Eastern China

**DOI:** 10.1186/1471-2334-10-323

**Published:** 2010-11-10

**Authors:** Aihua Sun, Xingli Fan, Ye Gu, Peng Du, Renxian Tang, Yafei Mao, Xuai Lin, Jie Yan

**Affiliations:** 1 Division of Basic Medical Microbiology, State Key Laboratory for Diagnosis and Treatment of Infectious Diseases, the First Affiliated Hospital of Zhejiang University, Zhejiang 310003, Hangzhou, China; 2 Faculty of Basic Medicine, Zhejiang Medical College, 310053 Zhejiang Hangzhou, China; 3 Department of Medical Microbiology and Parasitology, College of Medicine, Zhejiang University, Zhejiang 310058, Hangzhou, China; 4 College of Foreign Languages, Zhejiang Chinese Medicine University, Zhejiang 310053, Hangzhou, China; 5 Department of Medical Microbiology and Parasitology, Xuzhou Medical College, Jiangsu 221009, Xuzhou, China

## Abstract

**Background:**

Variations of *porB1A *and *porB1B *genes and their serotypes exist in *Neisseria gonorrhoeae *isolates from different geographical areas, and some site mutations in the *porB1B *gene correlate with drug resistance.

**Methods:**

The β-lactamase production of *N. gonorrhoeae *isolates was determined by paper acidometric test and nitrocefin discs. The *porB1A *and *porB1B *genes of 315 non-penicillinase-producting *N. gonorrhoeae *(non-PPNG) strains were amplified by PCR for sequencing to determine serotypes and site mutations. A duplex PCR was designed to simultaneously detect both *porB1A *and *porB1B *genes. Penicillin and tetracycline resistance was assessed by an *in vitro *drug sensitivity test.

**Results:**

Of the *N. gonorrhoeae *isolates, 31.1% tested positive for *porB1A *and 68.9% for *porB1B *genes. All the 98 *porB1A*^+ ^isolates belonging to IA6 serotype with either no mutation at the 120 and 121 sites (88.8%) or a D120G (11.2%) mutation and were no resistance to both penicillin and tetracycline. Among the 217 *porB1B*^+ ^isolates, 26.7%, 22.6% and 11.5% belonged to IB3, IB3/6 and IB4 serotypes, respectively. Particularly, two novel chimeric serotypes, IB3/6-IB2 and IB2-IB4-IB2, were found in 77 and 8 *porB1B*^+ ^isolates. Two hundred and twelve (97.7%) of the *porB1B*^+ ^isolates were presented G120 and/or A121 mutations with 163 (76.9%) at both sites. Interestingly, within the 77 *porB1B*^+ ^isolates belonging to IB3/6-IB2 serotype, 15 were discovered to possess novel deletions at both A121 and N122 sites. All the replacement mutations at these sites in PorB1B were correlated with resistance and the deletion mutation showed the highest resistance.

**Conclusion:**

*N. gonorrhoeae *isolates circulating in Eastern China include a sole PorB1A serotype (IA6) and five PorB1B serotypes. Multiple mutations in *porB1B *genes, including novel A121 and N122 deletions, are correlated with high levels of penicillin and tetracycline resistance.

## Background

Gonorrhoea caused by infection with *Neisseria gonorrhoeae *is a global sexually transmitted disease. In Chinese populations, gonorrhoea is the most common sexually transmitted disease which causes a serious public health problem [1-5].

The outer membrane of *N. gonorrhoeae *bears many proteins such as porins that have been studied in considerable detail [6-10]. Gonococcal porins are a group of outer membrane proteins that occur in large amounts on the surface [11-17]. PorB1A and PorB1B porins, which have 65-80% amino acid identity, share 60% of the total gonococcal porin proteins [12,18]. PorB1A and PorB1B are encoded by the same allele and any particular *N. gonorrhoeae *strain expresses either PorB1A or PorB1B [12,19,20]. PorB1A is present in 10-30% of *N. gonorrhoeae *isolates, while PorB1B occurs in 70-90% [21,22]. PorB1A and PorB1B are the serotyping basis of *N. gonorrhoeae *and mutations are more common in *porB1B *gene than in *porB1A *gene [19,22,23]. Many investigations revealed geographical diversity of the predominant *porB1A *and *porB1B *genotypes isolates in different areas [22,24-27]. Therefore, determination of *porB1A *and *porB1B *genotype distribution in *N. gonorrhoeae *isolates in different areas is very important for providing a high index of discrimination of different gonococcal strains, identifying the circulating strains and predominant serotypes, and tracking strain transmission in sexual contacts [28-32].

*N. gonorrhoeae *easily develops resistance to many antibiotics. Previous studies demonstrated that the replacement mutations at the 120 and 121 sites in PorB1B protein enable these strains to increase their resistance to penicillin and tetracycline [33,34]. However, replacement mutations at the 120 and 121 sites in PorB1A make little contribution to resistance against the two antibiotics [9,19]. Thus, identification of the two resistance-related site mutations in PorB1B sequences of *N. gonorrhoeae *isolates, and determination of the correlation between the site mutations and drug resistance are important for chemotherapy of gonorrhoea in the clinical setting. In the present study, we established a duplex polymerase chain reaction (PCR) system to rapidly confirm the *porB1A *and *porB1B *genes in *N. gonorrhoeae *isolates from Chinese patients. And the predominant serotypes based on *porB1A *and *porB1B *genotyping of the isolates circulating in Eastern China were determined by sequence analysis. Particularly, mutation at the 120 and 121 sites in PorB1B sequence from the non-penicillinase-producting *N. gonorrhoeae *(non-PPNG) isolates, as well as the correlation between the mutation patterns and the resistance to penicillin and tetracycline, were investigated.

## Methods

### Ethics statement

This research was conducted in accordance with the Declaration of Helsinki, and informed consent was obtained from all patients in this study for collection of clinical specimens to isolate *N. gonorrhoeae *strains, according to a protocol approved by the Ethics Committee of Zhejiang University.

### Bacterial strains and growth

The clinical *N. gonorrhoeae *strains were isolated from gonorrhea patients from 2005 to 2008 by the clinical laboratories of several hospitals in Zhejiang and Jiangsu provinces, China. All the gonococcal isolates were first identified by the hospital laboratories and subsequently rechecked by our laboratory using microscopy after Gram staining plus oxidase, catalase, and carbohydrate degradation tests [35]. The gonococci were cultured on GCB blood agar plates (bioMérieux, Co., Ltd, Shanghai, China) at 37°C in the presence of 5% CO_2 _for 24 h. *Staphylococcus aureus *ATCC25923, *Staphylococcus epidermidis *ATCC12228, *Streptococcus pyogenes *ATCC29212, *Escherichia coli *ATCC25922, *Pseudomonas aeruginosa *ATCC27543, *Klebsiella pneumoniae *ATCC700603, *Serratia marcescens *ATCC14041, *Enterobacter cloacae *ATCC13047 and *Proteus mirabilis *ATCC25933 were used to determine the specificity of duplex PCR, and were provided by the Chinese National Institute for Control of Pharmaceutical and Biological Products, and cultured in MH broth (bioMrieux) at 37°C for 24 h.

### β-lactamase detection

Paper acidometric test was applied by the clinical laboratories of hospitals to primarily detect the β-lactamase production of gonococcal isolates [5], and subsequently BBL(tm) Cefinase(tm) Paper Disc (BD, USA) based on nitrocefin coloration was used by our laboratory to further determine the production of gonococcal β-lactamase [36]. Among all the *N. gonorrhoeae *isolates, 315 strains were identified as non-PPNG that were used in this study.

### DNA preparation

The gonococci and the other bacteria were collected by centrifugation and washed twice with phosphate buffered saline (PBS). Genomic DNA of each precipitated strain was extracted with a bacterial genomic DNA extraction kit (BioColor Inc., Shanghai, China) and then dissolved in TE buffer for detecting the DNA concentration with ultraviolet (UV) spectrophotometry [37].

### Amplification of entire *porB1A *and *porB1B *genes

One pair of forward (porB1A/1B-F) and reverse (porB1A/1B-R) primers was used to amplify both the entire *porB1A *and *porB1B *genes in genomic DNA of the gonococcal isolates because they have the same nucleotide sequences at the 5' and 3' terminals (Table 1) [19,38,39]. A High Fidelity PCR Kit (TaKaRa Co., Ltd, Dalian, China), in which a Taq-Pfu mixture was used as the DNA polymerase, was used to amplify the two target genes. PCR was initiated by incubation at 94°C for 5 min, followed by 30 cycles at 94°C for 30 s, 54°C for 30 s and 72°C for 90 s to amplify each the two genes, and then incubation at 72°C for 10 min. The products in 1.5% ethidium bromide pre-stained agarose gel after electrophoresis were observed under UV light. The target products are predicted to be 981 bp (entire *porB1A *gene) and 1044 bp (entire *porB1B *gene).

**Table 1:** Primer sequences used in PCR for amplifying *porB1A *and *porB1B *genes

Primers	Sequences (5' to 3')*	Products and sizes
porB1A/1B	F: ATGAAAAAATCCCTGATTGCC	981 bp for entire *porB1A *gene, and 1044 bp for entire *porB1B *gene
	R: TTAGAATTTGTGGCGCAGAAC	
porB1A/1B-D	F1: GCCATTTGGCAGTTGGAACA	520 bp for partial sequence of porB1A gene, and 201 bp and 583 bp for partial sequence of porB1B gene
	F2: GATACGGCGAAGGCACTAAA	
	R: CTTCGGTTTGAGAGTTGTGC	

* F: forward primer, R: reverse primer

### Sequencing and genotyping

The PCR products of the entire *porB1A *and *porB1B *genes from the *N. gonorrhoeae *isolates were purified with a PCR products purification kit (BioColor) and then ligated into plasmid pMD-18-T to form recombinant pMD-18-T^porB1A ^and pMD-18-T^porB1B ^with a T-A cloning kit (TaKaRa). The inserted target segments in recombinant plasmids were sequenced using the double-stranded dideoxy chain termination method by Invitrogen Co., Ltd, Shanghai, China. The obtained sequencing data were analyzed as well as compared to the sequences of PorB1A and PorB1B serotypes published in GenBank using Clustalx software.

### Duplex PCR

The sequencing data of *porB1A *and *porB1B *genes from the *N. gonorrhoeae *isolates showed that these genes have over 80% identity at the 5' terminal sequences (1-240 bp segments in both genes) and approximately 90% identity at the 3' terminal sequences (714-981 bp segment in *porB1A *gene and 777-1044 bp segment in *porB1B *gene). However, a 63 bp segment in the middle region of *porB1B *gene that was absent in *porB1A *gene enabled us to design a specific forward primer only for detecting the *porB1B *gene. Thus, by using two different forward primers (20 pmol porB1A/1B-D-F1 and 20 pmol porB1A/1B-D-F2) and one common reverse primer (20 pmol porB1A/1B-D-R) (Table 1), a duplex PCR system was established to simultaneously detect the *porB1A *and *porB1B *genes in the gonococcal isolates. The total volume per duplex PCR was a 50 μl mixture in which 100 ng DNA template was used. The PCR was initiated by incubation at 94°C for 3 min, followed by 35 cycles at 94°C for 30 s, 54°C for 30 s and 72°C for 60 s, and then incubation at 72°C for 7 min. In agarose gel, the *porB1A *gene product presented a 520 bp fragment and the *porB1B *gene product showed two fragments of 201 bp and 583 bp.

### Drug sensitivity test

Susceptibility of the *N. gonorrhoeae *isolates to penicillin (bioMérieux) and tetracycline (bioMérieux) was assessed on GC agar plates (bioMérieux) by the standard proportion method [33,34]. Briefly, each freshly cultured isolate was suspended in GC broth to a density of 10^4^/μl, and 5 μl of gonococcal suspension was spotted onto GC agar plates containing increasing concentrations of penicillin or tetracycline (0.03, 0.06, 0.12, 0.25, 0.5, 1, 2, 4, 8, 16, 32, 64 and 128 μg/ml). After inoculation, the plates were incubated at 37°C in 5% CO_2 _for 24 h. The minimal inhibitory concentration (MIC) was defined as the minimum concentration of antibiotic at which no more than 5 colonies were observed after incubation. A MIC value ≥2 mg/L was considered as resistance to the antibiotics [40,41]. This test was repeated as three independent experiments.

## Results

### Sensitivity and specificity of duplex PCR

In our duplex PCR, *porB1A *and *porB1B *gene segments were effectually amplified when using a DNA concentration of *N. gonorrhoeae *as low as 10 ng, and negative results were shown when using 10 to 500 ng DNA templates of *S.aureus *ATCC25923, *S. epidermidis *ATCC12228, *S. pyogenes *ATCC29212, *E. coli *ATCC25922, *P. aeruginosa *ATCC27543, *K. pneumoniae *ATCC700603, *S. marcescens *ATCC14041, *E. cloacae *ATCC13047 and *P. mirabilis *ATCC25933.

### Distribution of *porB1A *and *porB1B *genes in *N. gonorrhoeae *isolates

**Figure 1:**
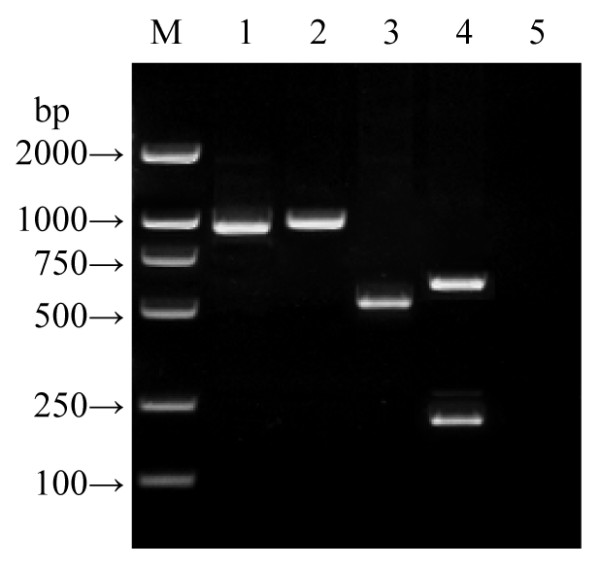
**Amplicons of entire or partial *porB1A *and *porB1B *genes**. Note: Lane M: DNA marker (BioColor); Lanes 1 and 2: amplicons of entire *porB1A *gene (981 bp) and *porB1B *gene (1044 bp), respectively; Lanes 3 and 4: amplicons of partial *porB1A *gene (520 bp) and partial *porB1B *gene (583 bp and 201 bp) by duplex PCR, respectively; Lane 5: blank control.

Of the 315 *N. gonorrhoeae *isolates tested, 31.1% (98/315) and 68.9% (217/315) were found by separate PCR to possess *porB1A *and *porB1B *genes, respectively. The duplex PCR established in this study accurately distinguished the *porB1A *and *porB1B *genes (Figure 1), and presented the same positive rates for *porB1A *(31.1%) and *porB1B *(68.9%) genes in the same gonococcal isolates.

### *porB1A *serotype in *N. gonorrhoeae *isolates

**Figure 2:**
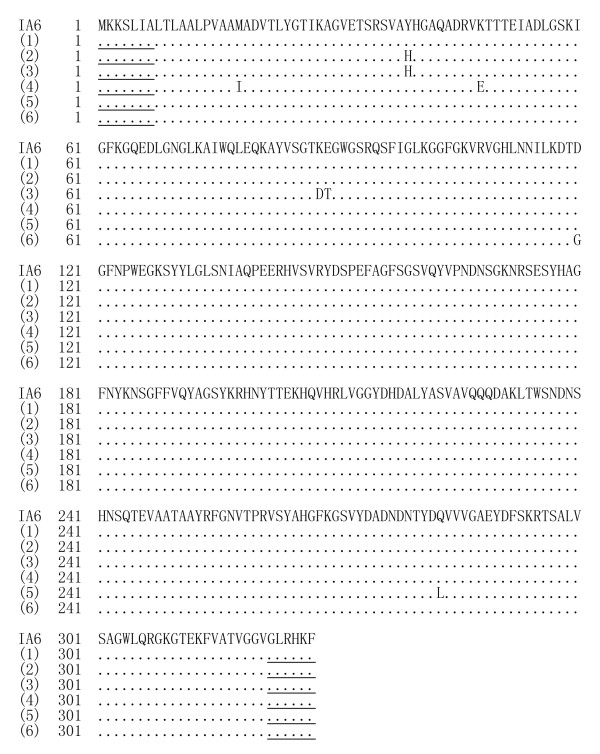
**Amino acid sequences from *porB1A *genes of *N. gonorrhoeae *isolates**. Note: (IA6): reported amino acid sequence of IA6 serotype (GenBank accession No: L19962); (1) and (2): PorB1A sequences from 56 and 23 gonococcal isolates, respectively; (3) to (5): PorB1A sequences from 8 gonococcal isolates; (6) PorB1A sequences from 11 gonococcal isolates. Underlined areas indicate positions of primers.

Compared to the *porB1A *sequences belonging to different PorB1A serotypes in GenBank, all the 98 *porB1A*^+ ^isolates had 99.47% to 100% nucleotide sequence identity and 99.04% to 100% amino acid sequence identity to the IA-6 serotype (GenBank accession No.: L19962, IA-6 serotype) (Figure 2) [19].

### *porB1B *serotypes in *N. gonorrhoeae *isolates

**Figure 3:**
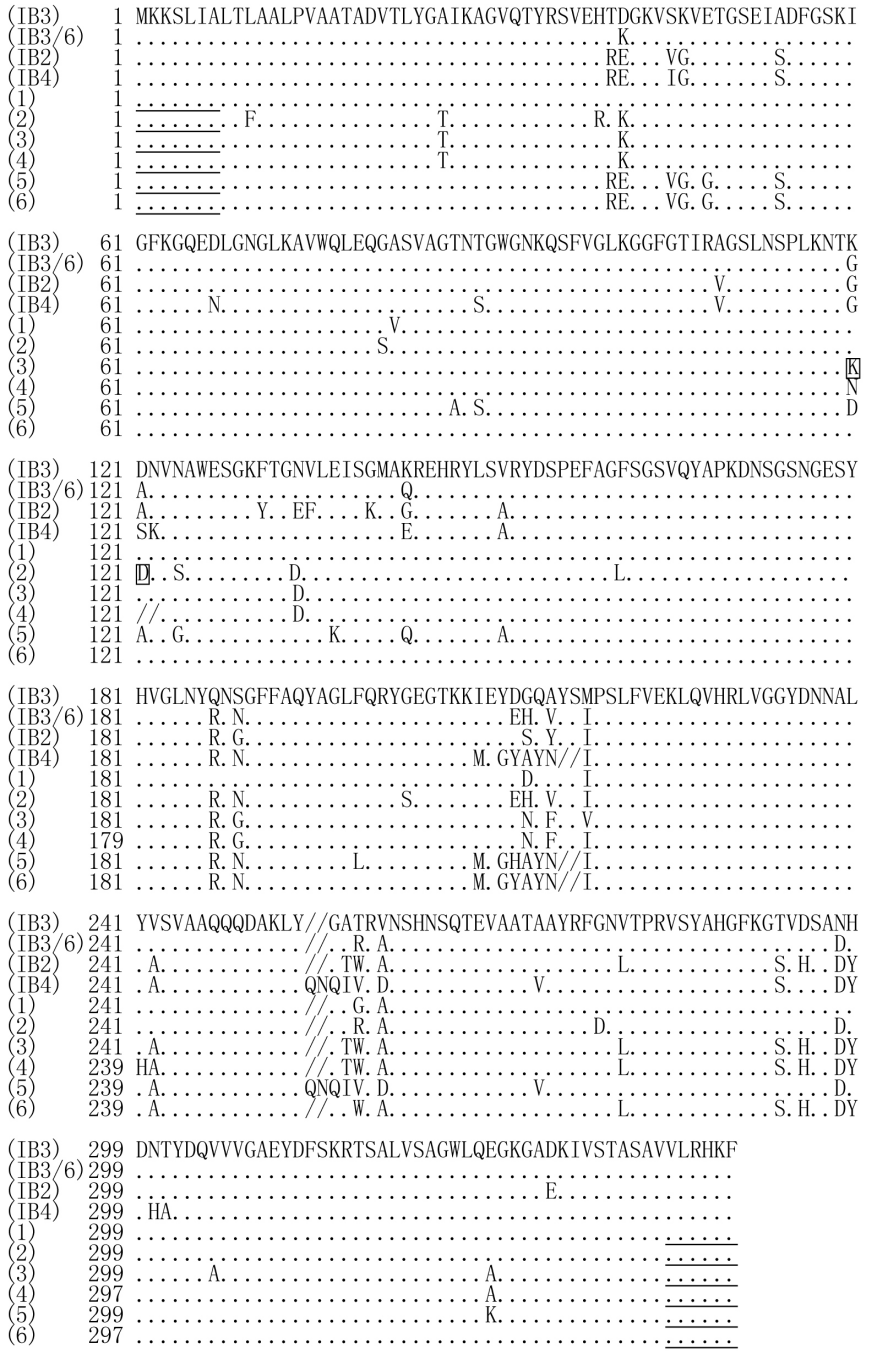
**Amino acid sequences from *porB1B *genes of *N. gonorrhoeae *strains**. Note: (IB3), (IB3/6), (IB2) and (IB4): reported PorB1B sequences belonging to serotypes IB3 (GenBank accession No: U75639), IB3/6 (GenBank accession No: U75641), IB2 (GenBank No: U75640) and IB4 (GenBank No: AF090797), respectively; (1): PorB1B sequence from 58 gonococcal isolates referring to IB3 serotype; (2): PorB1B sequence from 49 gonococcal isolates referring to IB3/6 serotype (D means the 27 isolates with A121D mutation, the 19 isolates with A121G mutation and the remaining 3 isolates with no mutation at the A121 site); (3): PorB1B sequence from 62 gonococcal isolates referring to IB3/6-IB2 chimeric serotype without A121 and N122 deletion (K means the 53 strains with G120K mutation and the remaining 9 strains with G120N mutation); (4): PorB1B sequence from 15 gonococcal isolates referring to IB3/6-IB2 chimeric serotype with A121 and N122 deletions; (5): PorB1B sequence from 25 gonococcal isolates referring to IB4 serotype; (6): PorB1B sequence from 8 gonococcal isolates referring to IB2-IB4-IB2 chimeric serotype. Underlined areas indicate the positions of primers. The signal "/" means lack of the amino acid residue.

Compared to the reported *porB1B *sequences belonging to different serotypes in GenBank [31,32], 26.7% (58/217), 22.6% (49/217) and 11.5% (25/217) of the 217 *porB1B*^+ ^gonococcal isolates belonged to serotypes IB3, IB3/6, and IB4, respectively. Some (35.5%; 77/217) of the *porB1B*^+ ^isolates were chimeras of the IB3/6 and IB2 serotypes (IB3/6-IB2 serotype) in which about half the sequences from the 5' terminal were like the IB3/6 serotype and the rest were like the IB2 serotype. Also, 3.7% (8/217) of the *porB1B*^+ ^isolates were chimeras of the IB2 and IB4 serotypes (IB2-IB4-IB2 serotype) in which the sequence in the middle was like the IB4 serotype but those at the two terminals were like the IB2 serotype (Figure 3).

### Mutations at the 120 and 121 sites in PorB1A and PorB1B

Mutations at the 120 and 121 sites in PorB1A and PorB1B sequences from all 315 *N. gonorrhoeae *isolates are listed in Table 2. In the 98 *porB1A*^+ ^isolates, 88.8% (87/98) had D120 and G121 in the PorB1A sequences which is identical to those in the reported PorB1A sequence belonging to the IA-6 serotype (GenBank accession No.: L19962) [19], and the remaining 11 *porB1A*^+ ^isolates had a D120G mutation alone (Figure 2). In the 217 *porB1B*^+ ^gonococcal isolates, only 2.3% (5/217, three belonging to the IB3/6 serotype and two belonging to the IB3 serotype) had no mutation at the 120 and 121 sites (G120 and A121), while 97.7% (212/217) showed various mutations at the sites. Particularly, in the 212 *porB1B*^+ ^isolates with the site mutations, 15 isolates (6.9%, 15/217) belonging to the IB3/6-IB2 serotype had a deletion of the amino acid residues at both 121 and 122 sites (Figure 3).

**Table 2:** G120 and A121 mutations in PorB1A and PorB1B of *N. gonorrhoeae *isolates*

Serotypes	Cases (n)	Site mutation patterns (n)							
		
		G120K/A121D	G120K/A121G	G120N/A121D	G120D/A121G	G120D	G120K	G120N	A121G
IB3	56	56	0	0	0	0	0	0	0
IB3/6	46	27	19	0	0	0	0	0	0
IB4	25	0	0	0	0	25	0	0	0
IB3/6-IB2	62	44	0	9	0	0	9	0	0
IB3/6-IB2^#^	15	0	0	0	0	0	0	15	0
IB2-IB4-IB2	8	8	0	0	0	0	0	0	0
IA6	98	0	0	0	87	0	0	0	11
Total	310	135	19	9	87	25	9	15	11

*: 5 isolates (3 for IB3/6 and 2 for IB3 serotypes) without G120 and A121 mutations not included. G, K, A, D and N indicate glycine, lysine, aspartic acid and asparagine, respectively. ^#^: isolates with deletions of both A121 and N122.

### Correlation between resistance and mutations at 120 and 121 sites in PorB1A and PorB1B

The MICs of penicillin and tetracycline for all the 98 *porB1A*^+ ^gonococcal isolates were 0.06-1 mg/L (<2 mg/L), while the MICs for both antibiotics in the 5 *porB1B*^+ ^gonococcal isolates with no mutations at the 120 and 121 sites in PorB1B were 0.12-0.5 mg/L (<2 mg/L). However, the MICs of penicillin and tetracycline in the 197 *porB1B*^+ ^isolates with replacement mutations at the 120 and/or 121 sites were 2-8 mg/L and 2-16 mg/L, respectively, and the 15 *porB1B*^+ ^isolates with deletion mutations at both the 121 and 122 sites had the highest resistance to penicillin (MICs = 4-8 mg/L) and tetracycline (both MICs = 4-16 mg/L) (Table 3). According to the MIC value (≥2 mg/L) that defines resistance to penicillin and tetracycline, all the *porB1A*^+ ^isolates and the 5 *porB1B*^+ ^isolates without site mutations were no resistance to both antibiotics, while all the remaining 212 *porB1B*^+ ^isolates with different site mutations were penicillin and tetracycline resistant.

**Table 3:** MICs of penicillin and tetracycline for *N. gonorrhoeae *isolates

Site mutations*	Serotypes	Cases (n)	MICs (mg/L)	
			
			Penicillin	Tetracycline
G120K/A121D	IB3	56	2-4	2-8
	IB3/6	27	2-4	2-8
	IB3/6-IB2	44	2-4	2-8
	IB2-IB4-IB2	8	2-4	2-4
G120K/A121G	IB3/6	19	2-4	2-8
G120N/A121D	IB3/6-IB2	9	2	2-4
G120D	IB4	25	2-4	2-8
G120K	IB3/6-IB2	9	2-4	2-8
G120N/A121 and N122 deletion	IB3/6-IB2	15	4-8	4-16
G120G/A121A	IB3/6	3	0.12	0.25
	IB3	2	0.25	0.25
D120D/G121G	IA6	87	0.06-0.5	0.06-0.5
D120G/G121G	IA6	11	0.12-0.25	0.12-1

*: G, K, A, D and N indicate glycine, lysine, aspartic acid and asparagine, respectively.

## Discussion

The porins of *N. gonorrhoea *stimulate the host immune system to produce specific antibodies which activate the complement system and promote phagocytosis to eliminate the invading gonococci [9,42]. Furthermore, gonococcal PorB1A and PorB1B are closely associated with transmission of gonorrhea [38,42], and mutations at the 120 and 121 sites in PorB1B are related to drug resistance [9,19,33,34]. Thus, determination of the predominance of PorB1A and PorB1B serotypes and drug resistance-associated mutations in the *porB1B *gene in different areas is important for serological diagnosis and chemotherapy of gonorrhoea.

For rapid and convenient discrimination of *porB1A *and *porB1B *genes in *N. gonorrhoeae *isolates, we designed a duplex PCR system by which both genes were simultaneously detectable. The results indicated that the duplex PCR accurately recognizes the *porB1A *and *porB1B *genes in all the 315 tested isolates with high sensitivity and specificity. By using the duplex PCR, we found 31.1% of the 315 isolates tested were positive for the *porB1A *gene and the *porB1B *gene was detectable in 68.9% of the isolates. This *porB1A*^+ ^and *porB1B*^+ ^proportion in *N. gonorrhoeae *isolates is close to that of previous reports [21,22].

The gonococcal *porB1B *gene differs from the *porB1A *gene in having many more mutations in its sequence [19,38,39]. Among the 217 *porB1B*^+ ^isolates in this study, 77 (35.5%), 58 (26.7%), and 49 (22.6%) isolates belonged to the IB3/6-IB2, IB3 and IB3/6 serotypes, and only 25 (11.5%) and 8 (3.7%) referred to the IB4 and IB2-IB4-IB2 serotypes, respectively. These data indicate that the predominant serotypes of *porB1B*^+ ^*N. gonorrhoeae *in Eastern China are IB3/6-IB2, IB3 and IB3/6 (84.8%, 184/217), which quite differs from the IB2, IB4 and IB3 reported as the predominant serotypes in nations in Europe and North America [19,22-24]. Bash and colleagues (2005) showed that the *porB1B *gene has a high recombinant mutation frequency compared to the *porB1A *gene [43]. In our study, two novel recombinant mutations formed IB3/6-IB2 and IB2-IB4-IB2 chimeric serotypes, which also indicates a high frequency of recombination among different gonococcal *porB1B *genes. There have been reports of *N. gonorrhoeae *strains that react to both PorB1A and PorB1B antibodies, raising the possibility of mosaicism between *porB1A *and *porB1B *alleles [44,45]. However, no such mosaicism was found in all the *N. gonorrhoeae *isolates tested in this study.

Olesky et al. (2002) used the site mutation technique to generate mutants at the 120 and 121 sites in PorB1B, and found stronger resistance to penicillin and tetracycline in the G120K, G120D/A121D, G120K/A121R and G120P/A121P mutants [46]. Among the 217 *porB1B*^+ ^isolates in this study, only five had no mutations at the 120 and 121 sites, while the other 212 had various mutations at these sites, in which 76.9% (163/212) had double-site mutations (G120K/A120D, G120K/A121G and G120N/A121D in 135, 19 and 9 isolates). All the 212 *porB1B*^+ ^isolates had a mutation at the G120 site replaced with lysine (K) (76.9%, 163/212), aspartate (D) (11.8%, 25/212) or asparagine (N) (11.3%, 24/212). On the other hand, 76.9% of the 212 *porB1B*^+ ^strains had a mutation at the A121 site replaced with aspartate (D) (88.3%, 144/163) or glycine (G) (11.7%, 19/163). However, the G120R, G120P, A121H and A121P mutations described by Olesky *et al *were not found, suggesting differences in the amino acids replacement by natural and artificial mutations. Particularly, in this study we found that 15 gonococcal isolates had A121 and N122 deletion mutations in PorB1B that had not previously been reported.

Many previous data revealed that plasmid-mediated resistance of *N. gonorrhoeae *confer the very high MICs of penicillinand tetracycline [47-49], while chromosomally mediated resistance such as the site mutations at G120 or A121 in gonococcal PorB1B contributes to the moderate higher MICs of the two antibiotics [33,34]. According to our drug sensitivity tests for the non-PPNG isolates, the G120K/A121D, G120K/A121G, G120N/A121D, G120D and G120K natural *porB1B *mutants also had stronger resistance to penicillin and tetracycline than *porB1B*^+ ^isolates with no G120 and A121 mutations (Table 3). Interestingly, the 15 *porB1B*^+ ^isolates with A121 and N122 deletions had the highest MICs, which hints at a greater influence of the A121 deletion on resistance than any of the replacement mutations. This resistance relating to chromosomally-mediated site mutations in PorB1B has been shown in many previous reports [33,46,50-52]. However, in these reports the PorB1B mutation-associated resistance only noted MICs of penicillin and tetracycline in the range of 2-8 μg/ml. Previous data revealed that site mutations in the *penA*, *mtrR*, *ponA*, and *rpsJ *genes also participate in chromosomally-mediated gonococcal resistance to penicillin and tetracycline [47,53-55]. Thus, synergistic action of site mutations in multiple chromosomal genes associated with resistance in *N. gonorrhoeae *isolates is an important subject for investigation. Such action may be responsible for the high resistance to both antibiotics found in this study.

We found that all the tested *porB1A*^+ ^gonococcal isolates were no resistance to both penicillin and tetracycline, whereas 97.7% (212/217) of the *porB1B*^+ ^isolates had site mutation-based resistance in the PorB1B sequences. The high proportion (100%) of resistance-associated mutations at the 120 and 121 sites in PorB1B of *N. gonorrhoeae *isolates from other Chinese areas has also been reported [51,52,56]. Thus, the duplex PCR established in this study for rapid identification of *porB1A*^+ ^and *porB1B*^+ ^gonococci provides a useful tool for selecting antibiotics to treat gonorrhea in China.

## Conclusions

The duplex PCR system we designed simultaneously recognizes *porB1A *and *porB1B *genes in *N. gonorrhoeae *isolates with high sensitivity and specificity. *porB1B*^+ ^*N. gonorrhoeae *is the predominant genotype in Eastern China. All the *porB1A*^+ ^isolates had the conserved *porB1A *gene sequence belonging to the IA6 serotype alone. However, the *porB1B*^+ ^isolates had high site mutations in the *porB1B *sequence that resulted in five PorB1B serotypes. All the *porB1A*^+ ^isolates were no resistance to both penicillin and tetracycline. However, multiple penicillin and tetracycline resistance-related mutations such as G120K/A121D, G120K/A121G and G120N/A121D occurred in PorB1B isolates. A novel mutation due to deletion of both A121 and N122 in PorB1B is correlated with high resistance to penicillin and tetracycline.

## List of Abbreviations

STD: sexual transmitted disease; G: glycine; D: aspartic acid; K: lysine; R: arginine; P: proline; A: alanine; H: histidine; N: asparagine; PCR: polymerase chain reaction; DNA: deoxyribonucleic acid; PBS: phosphate buffered saline; TE: Tris-EDTA; Tris: trihydroxymethyl aminomethane; EDTA: ethylenediamine tetraacetic acid; UV: ultraviolet rays; MIC: minimal inhibitory concentration

## Competing interests

The authors declare that they have no competing interests.

## Authors' contributions

AS participated in the experimental design, obtained funding for the study, performed PCR and drafted the manuscript. XF supervised the collection of gonococcal isolates, and helped to draft the manuscript. PD analyzed the sequencing data. YG helped to draft the manuscript. RT helped to collect the gonococcal isolates. YM helped to perform PCR. XL carried out the drug sensitivity tests. JY participated in the experimental design and obtained funding for the study. All authors have read and approved the final manuscript.

## Pre-publication history

The pre-publication history for this paper can be accessed here:

http://www.biomedcentral.com/1471-2334/10/323/prepub
